# Artificial Intelligence and Anatomical Variation: A Narrative Review of Detection, Classification, and Educational Applications

**DOI:** 10.7759/cureus.116649

**Published:** 2026-09-21

**Authors:** Ahmed Mahgoub, Anaida Singh, Ayman Mahgoub, Deepak Sharma

**Affiliations:** 1 Anatomy, Tanta University Hospital, Tanta, EGY; 2 Medicine, St. George's University, St. George's, GRD; 3 Research and Development, ANOVA Health, Riyadh, SAU; 4 Basic Science, Lake Erie College of Osteopathic Medicine, Erie, USA

**Keywords:** anatomical variants, anatomical variation, anatomy education, artificial intelligence, deep learning, large language models, machine learning, surgical planning, vision-language models

## Abstract

Anatomical variation encompasses non-pathological differences in the number, morphology, position, course, or relationships of structures and is not synonymous with rarity. Such variation affects imaging, surgery, procedural safety, dentistry, and anatomy education. This structured narrative review maps artificial intelligence (AI) applications that detect, classify, or segment variable anatomy across organ systems and distinguishes variant-specific evidence from general structure recognition. PubMed/MEDLINE, Google Scholar, publisher records, and preprint servers were searched iteratively on September 4, 2026, and updated on September 7, 2026, with citation chaining. The synthesis includes 14 principal empirical reports identified by this process: nine direct clinical imaging studies, three variation-relevant mapping studies, one direct multimodal preprint benchmark, and one contextual educational evaluation. Direct clinical evidence is concentrated in dental and cranio-maxillofacial imaging, with additional studies of lumbosacral transitional vertebrae and pulmonary, hepatic, or portal venous patterns. Performance is task-, dataset-, and prevalence-dependent; external validation, calibration, and transparent handling of correlated observations are uncommon. Segmentation performance does not establish named-variant classification, and evidence from anatomical proximity should not be transferred to untested variants. Educational outcome evidence is preliminary. Current AI may support narrowly specified tasks, but the identified literature does not establish a general, transferable understanding of human anatomical diversity. Progress requires explicit taxonomies, patient-level partitions, representative class distributions, clinically defensible reference standards, external validation, and dedicated educational evaluation.

## Introduction and background

Anatomical variation is an expected feature of biological diversity. In this review, anatomical variation is the broad concept of non-pathological differences in the number, morphology, position, course, or relationships of structures. A normal variant is a recognized configuration that differs from the most common pattern but is not itself a disease. Atypical anatomy is used only when an original study or benchmark contrasts common configurations with less common or anomalous presentations; it is not treated automatically as synonymous with a normal variant. Rare anatomy is reserved for configurations supported by prevalence evidence as uncommon. Congenital anomalies such as polydactyly and horseshoe kidney fall outside the principal clinical imaging scope unless an included source explicitly evaluates them within an anatomical variation framework.

Anatomical variants can alter operative planning, radiological interpretation, regional anesthesia, endodontic treatment, and educational resources [1,2]. Artificial intelligence (AI) systems address different tasks: detection locates a target; classification assigns a prespecified category; segmentation delineates a structure; and multimodal systems combine images with language. General recognition or segmentation of a structure does not demonstrate that a model identifies a named variant. Likewise, performance on typical anatomy cannot be assumed to transfer to underrepresented configurations. Systems trained predominantly on common patterns may reproduce the statistical norm while overlooking or mischaracterizing less frequent anatomy [3-5].

Prior reviews have examined anatomical variation in practice and education [2], AI in anatomy teaching and large language model use [6-8], anatomical structure recognition in surgery [9,10], vascular AI [11], and root and canal anatomy [12]. These reviews have not integrated variant-focused clinical imaging evidence across anatomical systems with educational and multimodal AI applications. This review therefore aims to characterize direct and variation-relevant AI applications, distinguish variant-specific evidence from adjacent structure- or disease-focused work, and identify clinical, educational, and methodological priorities. The central question is what evidence supports AI use when anatomy departs from the common configuration.

## Review

Methods

A narrative design was selected because the evidence spans heterogeneous classification, segmentation, clinical planning, educational, and multimodal studies that cannot be pooled meaningfully. The objective was a structured cross-system synthesis, not a systematic prevalence estimate. No protocol was registered, Preferred Reporting Items for Systematic Reviews and Meta-Analyses (PRISMA) compliance is not claimed, and screening and extraction were not independently duplicated.

A structured, iterative search was conducted on September 4, 2026, and updated on September 7, 2026. Sources were PubMed/MEDLINE, Google Scholar, publisher records, arXiv, and medRxiv; reference lists of relevant reviews and empirical reports were citation-chained. Table 1 documents the query blocks used. Google Scholar supported exact-title, DOI, author, and named-target discovery and bibliographic verification; it was not used as a reproducible database search, and the number of results or pages examined was not retained. Record-level yields, duplicate counts, and a full-text exclusion log were also not retained and were not reconstructed retrospectively.

**Table 1:** Structured, iterative search framework Searches were run on September 4, 2026, and updated on September 7, 2026. The table documents the framework used but does not imply systematic review completeness or full reproducibility.

Source/search block	Search expression or procedure	Purpose
PubMed/MEDLINE: broad variation	("artificial intelligence" OR "machine learning" OR "deep learning" OR "convolutional neural network" OR "large language model" OR "vision-language model" OR ChatGPT) AND ("anatomical variation" OR "anatomic variation" OR "anatomical variant" OR "anatomic variant" OR "normal variant")	Broad cross-system identification
Dental and sinonasal targets	("artificial intelligence" OR "machine learning" OR "deep learning" OR "convolutional neural network" OR "large language model" OR "vision-language model" OR ChatGPT) AND ("root morphology" OR "root canal" OR "C-shaped canal" OR "three-rooted mandibular molar" OR "mandibular canal" OR "accessory maxillary ostium" OR "sinonasal variant" OR "Onodi cell" OR "Haller cell" OR "anterior ethmoidal artery")	Direct dental/sinonasal and canal mapping studies
Skeletal, thoracic, abdominal targets	("artificial intelligence" OR "machine learning" OR "deep learning" OR "convolutional neural network" OR "large language model" OR "vision-language model" OR ChatGPT) AND ("lumbosacral transitional vertebra" OR "pulmonary vessel" OR "hepatic vein" OR "portal vein" OR "renal artery" OR "accessory renal artery" OR "visceral variant")	Vertebral and vascular evidence; renal studies used only for boundaries
Peripheral nerve targets	("artificial intelligence" OR "machine learning" OR "deep learning" OR "convolutional neural network" OR "large language model" OR "vision-language model" OR ChatGPT) AND ("peripheral nerve variation" OR "nerve variant" OR "regional anesthesia anatomy recognition")	Named variants and adjacent annotation literature
Education and foundation models	("large language model" OR "vision-language model" OR ChatGPT OR "artificial intelligence") AND ("anatomy education" OR "anatomical variation" OR "atypical anatomy" OR "normal variant")	Educational evaluations and typical-versus-atypical benchmarks
Supplementary discovery	Exact-title, DOI, author, and named-target searches; arXiv and medRxiv version checks; citation chaining	Discovery, metadata verification, and version checking

English-language reports published from January 2018 through September 7, 2026, were eligible when they examined human anatomy or medically oriented human image data and prespecified a normal variant, evaluated a spatially variable structure, or directly informed interpretation of such work. Earlier sources were retained for foundational context. Reviews supported orientation and citation chaining but were not treated as primary validation evidence. Animal, cellular, pathology-only, and non-AI prevalence studies were excluded from the principal synthesis. The English-language restriction reflected the team's inability to verify technical terminology, classifications, and methods reliably in other languages; automated translation alone was not considered sufficient for definitive extraction.

Evidence categories were operationalized before the final synthesis. Direct evidence required an explicit variant target and at least one of the following: a named variant, a branching or configuration taxonomy, a typical-versus-atypical comparison, or variant-level classification/performance. Variation-relevant evidence mapped or segmented a structure known to vary but did not classify a named variant or report variant-level performance. Contextual evidence informed rationale, boundaries, or interpretation without empirically testing variant recognition. Pulmonary and hepatic/portal studies were direct because they classified explicit branching or configuration patterns; mandibular canal studies were variation-relevant because they primarily traced the canal without named-variant classification.

The 14 principal empirical reports were the studies used in the main synthesis: nine direct clinical imaging studies, three variation-relevant mapping studies, one direct multimodal preprint benchmark, and one contextual educational evaluation. All nine direct clinical imaging reports discussed in this review appear in a table below. Other citations provide background, secondary synthesis, methodological guidance, or boundary evidence. Selection and classification were author-led; uncertain cases were resolved through repeated comparison with the operational definitions rather than independent duplicate review.

For each principal report, the review extracted the anatomical target and taxonomy, intended function, number of patients and other units, class distribution, reference standard, partitioning approach, validation setting, performance metrics, publication status, and limitations. Missing information was recorded as not reported (NR) or not inferred. Study-level appraisal domains were informed by QUADAS-2, PROBAST, and CLAIM [13-15] and included unit independence, class balance, reference standards, patient-level splitting, leakage risk, clustered observations, confidence intervals, calibration, and applicability. No overall quality score was assigned.

Synthesis followed a structured narrative approach informed by Synthesis Without Meta-analysis (SWiM) principles [16]. Studies were grouped by intended AI function and anatomical domain and then compared by target definition, dataset composition, unit of analysis, reference standard, validation design, and outcome. Conflicting results were interpreted in relation to these differences rather than pooled. No meta-analysis or new inferential analysis was undertaken because the clinical and methodological heterogeneity made quantitative pooling inappropriate.

As a manuscript-level reporting check, the review was also assessed against the six SANRA domains [17]. This author-led check improved reporting but did not constitute independent validation or convert the work into a systematic review.

Evidence categories

The operational criteria used to distinguish direct, variation-relevant, and contextual evidence are summarized in Table 2.

**Table 2:** Operational evidence categories used in the narrative synthesis

Category	Minimum operational requirement	Interpretive boundary
Direct	Explicit variant target plus named taxonomy, configuration class, typical-versus-atypical comparison, or variant-level performance	Supports only the specified target, population, modality, and workflow
Variation-relevant	Maps/segments a variable structure without named-variant classification or variant-level performance	Does not establish recognition of a clinically named variant
Contextual	Informs rationale, education, methods, or boundaries without empirical variant recognition	Cannot validate variant detection capability

Evidence synthesis

Principal Evidence Map

The identified evidence is concentrated in dental and cranio-maxillofacial imaging. Direct studies also address lumbosacral transitional vertebrae and pulmonary, hepatic, or portal venous configurations. Mandibular canal studies evaluate mapping rather than named-variant classification. Renal disease/workflow models and a non-AI accessory artery synthesis provide boundary and prevalence context but do not validate accessory artery detection [18-20]; regional anesthesia annotation literature similarly identifies ground-truth challenges without testing a named nerve variant [21]. One preprint benchmark compares typical and atypical anatomy across systems, while direct educational outcome studies remain largely absent. Table 3 presents the intended use, taxonomy, independent units, reference standard, evaluation design, and publication status for the principal reports.

**Table 3:** Principal empirical reports concerning AI and anatomical variation AI: artificial intelligence; AEA: anterior ethmoidal artery; AUC: area under the receiver operating characteristic curve; CBCT: cone-beam CT; IoU: intersection over union; LSTV: lumbosacral transitional vertebra; NR: not reported in the available study description; VLM: vision-language model Classification and segmentation metrics answer different questions and should not be compared directly. Predictive values are clinically interpretable only under an applicable prevalence.

Study	Function and taxonomy	Independent units/class balance	Reference standard	Evaluation and result	Status/key limitation
Kwak et al. 2025 [22]	Classify LSTV; binary LSTV/normal	3,116 patients/radiographs; test 313 (195 LSTV, 118 normal)	Whole-spine imaging, CT, or MRI	Independent internal test; 76.4% accuracy, 85.1% sensitivity, 61.9% specificity, AUC 0.84	Peer-reviewed; single health system development
Hiraiwa et al. 2019 [23]	Classify single vs. extra distal root	400 patients; 760 teeth	CBCT labels for panoramic images	Internal; 86.9% accuracy	Peer-reviewed; correlated bilateral teeth not fully addressed
Jeon et al. 2021 [24]	Classify C-shaped canal	1,020 patients; 2,040 teeth; 887 positive (43.5%)	Radiologist-reviewed CBCT	80/20 internal split; 95.1% accuracy, 92.7% sensitivity, 97% specificity	Peer-reviewed; patient-level split NR
Kwak et al. 2020 [25]	Segment mandibular canal; no variant taxonomy	Unit count NR in review extraction; CBCT	Expert canal annotations	Internal; reported global accuracy up to 0.99	Peer-reviewed; segmentation metric, not variant accuracy
Yang et al. 2024 [26]	Classify C-shaped canal configuration	805 patients; four institutions/two countries	CBCT-supported labels	Four external datasets; AUC 0.797-0.945	Peer-reviewed; performance varied by dataset/resolution
Jin et al. 2024 [27]	Classify two- vs. three-rooted molars	730 patients; 1,444 teeth	CBCT labels for panoramic images	Internal; 87.5% accuracy, AUC 0.907	Peer-reviewed; population-specific class distribution
Shetty et al. 2025 [28]	Detect accessory maxillary ostium	856 CBCT scans; 454 cropped sections; test n=34 images	Expert CBCT assessment	Internal test; 81% accuracy, AUC 0.87	Peer-reviewed; derived images are not from independent patients
Rita et al. 2026 [29]	Classify free-floating AEA, Onodi, and Haller cells	122 patients; CT	Expert imaging labels	Repeated patient-wise cross-validation; balanced accuracy 63.7-77.8%	Peer-reviewed; no external validation
Dhar and Yu 2021 [30]	Trace mandibular canal centerline; no variant taxonomy	CBCT; independent patient count NR	Generated centerline ground truth	Internal experimental evaluation; voxel and distance metrics	Preprint; clinical validation limited
Usman et al. 2022 [31]	Segment mandibular canal; no variant taxonomy	500 internal + 15 public CBCT scans	Expert/benchmark masks	Mostly internal; Dice 0.748, mean IoU 0.80	Peer-reviewed; segmentation does not establish variant recognition
Chen et al. 2022 [32]	Recognize pulmonary arterial/venous patterns for planning	27 surgical cases; three institutions	Senior surgeons using CT, 3D reconstruction, and operative observation	Independent multicenter pilot; case-wise accuracy 82.8%	Peer-reviewed; small, selected sample
Xie et al. 2024 [33]	Classify hepatic/portal trunk and branch anatomy	170 development + 178 external test patients	Imaging/clinical workflow reference	Two external datasets; 81.46-98.88% accuracy by vessel	Peer-reviewed; task embedded in volumetry workflow
Mayer et al. 2025 [5]	Typical vs. atypical multimodal benchmark	200 matched image-question pairs; seven modalities	Curated expert-annotated benchmark	25 VLMs; marked typical-atypical performance gap with 95% bootstrap CIs	Preprint; curated benchmark, not clinical deployment
Totlis et al. 2023 [4]	Contextual educational evaluation	18 structured ChatGPT questions	Expert qualitative assessment	No dedicated variant outcome	Peer-reviewed; conversational evaluation

Structured Methodological Appraisal

Table 4 summarizes the structured methodological appraisal of the principal reports across unit independence, class distribution, reference standards, data partitioning, validation, uncertainty, and applicability.

**Table 4:** Structured methodological appraisal of the principal reports Domains were informed by QUADAS-2, PROBAST, and CLAIM but adapted descriptively to heterogeneous classification, segmentation, planning, and benchmark studies; no overall score was assigned. CV: cross-validation; N/A: not applicable; NR: not reported

Study	Unit independence	Class/prevalence	Reference standard	Partition/leakage	Validation	Uncertainty/applicability
Kwak et al. 2025 [22]	Patient/radiograph aligned	Reported	Supported imaging	Patient split reported	Internal only	CIs/calibration NR
Hiraiwa et al. 2019 [23]	Patients vs. derived units clarified where available	Partly reported	Study-specific	Unclear or internal	No external validation	Clustering/CIs/calibration often NR
Jeon et al. 2021 [24]	Multiple teeth/patient	Reported	CBCT	Patient-level split unclear	Internal	CIs/calibration NR
Kwak et al. 2020 [25]	Patients vs. derived units clarified where available	Partly reported	Study-specific	Unclear or internal	No external validation	Clustering/CIs/calibration often NR
Yang et al. 2024 [26]	Patients reported	Study-specific	CBCT-supported	External datasets	External multicenter	Calibration NR
Jin et al. 2024 [27]	Patients vs. derived units clarified where available	Partly reported	Study-specific	Unclear or internal	No external validation	Clustering/CIs/calibration often NR
Shetty et al. 2025 [28]	Patients vs. derived units clarified where available	Partly reported	Study-specific	Unclear or internal	No external validation	Clustering/CIs/calibration often NR
Rita et al. 2026 [29]	Patients reported	Target-specific	Expert CT labels	Patient-wise CV	Internal CV	External validation/calibration NR
Dhar and Yu 2021 [30]	Patients vs. derived units clarified where available	Partly reported	Study-specific	Unclear or internal	No external validation	Clustering/CIs/calibration often NR
Usman et al. 2022 [31]	Patients vs. derived units clarified where available	Partly reported	Study-specific	Unclear or internal	No external validation	Clustering/CIs/calibration often NR
Chen et al. 2022 [32]	Cases reported	Selected pilot	Operative-supported	Independent cases	Multicenter pilot	Small sample; wide uncertainty likely
Xie et al. 2024 [33]	Patients reported	Vessel-specific	Workflow reference	External patients	External datasets	Calibration NR
Mayer et al. 2025 [5]	Matched image pairs	Curated balanced design	Expert benchmark	Pair-based evaluation	Benchmark only	95% bootstrap CIs; clinical calibration N/A
Totlis et al. 2023 [4]	Questions, not patients	N/A	Expert judgement	N/A	Contextual	No learner outcomes

Clinical Imaging Evidence

The LSTV study used 3,116 patient-level radiographs and an independent internal test set of 313, including 195 LSTV-positive and 118 normal cases [22]. Accuracy was 76.4%, but the accompanying 85.1% sensitivity and 61.9% specificity show why a single headline value is insufficient. The lower specificity implies substantial false-positive classification in this test set. The study supports feasibility for one radiographic task but remains internally validated and does not establish performance across other vertebral variants, modalities, institutions, or populations.

Dental studies classified extra roots and C-shaped canals using CBCT-supported reference labels [23,24,26,27,34]. The clearest advance is multicenter external validation for C-shaped canals, although performance varied across datasets and image resolution [26]. These reports demonstrate discrete classification rather than segmentation alone, but several analyze two teeth per patient; patient-level partitioning and adjustment for correlated observations are not always clear. Prevalence and population differences also limit direct comparison of accuracy and predictive values.

Sinonasal work addresses accessory maxillary ostia and surgically relevant CT variants [28,29]. The accessory ostium test comprised only 34 cropped images, while the three-target CT study used repeated patient-wise cross-validation in 122 patients and showed target-dependent balanced accuracy. These studies illustrate how derived image counts can exaggerate apparent sample size and how performance may vary even within one region.

Mandibular canal studies primarily trace or segment a variable structure [25,30,31]. Dice, intersection over union, and voxel accuracy describe spatial overlap; they do not establish classification of bifid canals, anterior loops, or other named variants. Pulmonary and hepatic/portal studies used explicit vessel pattern or branch classifications and included multicenter or external evaluation [32,33], but one was a small selected pilot and the other embedded classification within a broader planning workflow.

Multimodal and Educational Evidence

The AdversarialAnatomyBench preprint directly compared matched typical and atypical image-question cases across seven modalities and reported a substantial performance gap across 25 VLMs, with bootstrap confidence intervals [5]. It provides direct evidence of anatomical prior bias within a curated perception benchmark, not proof of clinical harm or educational effectiveness. Totlis et al. provided a qualitative educational evaluation in which variant-related responses were inadequate without systematic classifications [4]. Broader large language model evaluations address conventional anatomy performance but do not provide variant-specific learner outcomes [35]. Together, these sources justify further study but not claims of established educational benefit.

Discussion

Principal Findings

AI can support narrowly defined variant detection or classification tasks, particularly in dental and cranio-maxillofacial imaging. Evidence is nevertheless target-, dataset-, prevalence-, and workflow-specific. External validation is uncommon, and direct typical-versus-atypical testing is represented chiefly by one preprint benchmark. Anatomical variation is therefore a useful stress test for AI, but the identified evidence does not establish a general ability to handle human anatomical diversity.

Methodological Limitations of Existing Studies

Study size must be interpreted at the patient level. Counts of teeth, vessels, images, slices, cropped regions, or augmented samples do not represent independent biological observations. Failure to partition by patient can cause leakage, while failure to account for repeated observations can narrow confidence intervals. Class balance and natural prevalence are also essential: accuracy and predictive values vary with prevalence, area under the receiver operating characteristic curve (AUC) measures discrimination rather than calibration, and Dice or intersection over union measures spatial overlap rather than named-variant recognition. Across the evidence map, confidence intervals, calibration, threshold selection, clustering adjustments, and complete patient-level partitioning were often unreported.

Reference standards ranged from CBCT-supported dental labels and operative pulmonary observations to expert contours and curated benchmark answers. External validation across institutions, scanners, populations, and prevalence conditions remains the exception. These limitations support cautious interpretation rather than cross-study ranking.

Clinical and Educational Implications

Clinically, a model should be used only for its specified target, input, population, and workflow. Anatomical proximity does not justify capability transfer: a renal artery stenosis model does not thereby detect accessory renal arteries, and a canal segmenter does not necessarily classify named canal variants. AI should assist, not autonomously exclude, a variant unless that use has been validated directly.

Current educational evidence is limited to a qualitative conversational evaluation and the multimodal preprint benchmark [4,5]. Practical recommendations, such as expert verification of variant definitions, prevalence, laterality, population context, and clinical relevance, are therefore interpretive safeguards rather than proven interventions. Structured prompting, curated retrieval, learner-model comparisons, variant-specific question banks, automation bias assessment, and curriculum integration are proposed research priorities, not evidence-supported educational treatments; the rationale for teaching variation is established, but these AI-mediated approaches still require direct evaluation [36].

Research Priorities

Future studies should prespecify clinically meaningful variant targets and taxonomies; report independent patients, positive cases, natural prevalence, correlated observations, and augmentation; use patient-level partitions; and provide sensitivity, specificity, confidence intervals, calibration, and threshold procedures where applicable. External multicenter testing and comparison with relevant clinicians should evaluate whether assistance improves decisions rather than merely reproducing labels. Educational research should measure learner outcomes and automation bias using peer-reviewed, provenance-transparent benchmarks.

Limitations of This Review

This is a structured narrative review, not a systematic or scoping review. Complete executable strategies for every supplementary search, record yields, duplicate counts, Google Scholar screening depth, full-text exclusion reasons, and an excluded studies log were not retained. Selection and extraction were not independently duplicated, no protocol was registered, and the appraisal was adapted rather than a formal design-specific risk-of-bias assessment. English-language restriction may have excluded evidence from some populations and regions. The 14 principal reports should therefore be interpreted as an evidence map identified by the stated process, not an exhaustive global inventory. Heterogeneity in targets, units, reference standards, validation, and metrics precluded quantitative pooling. One central cross-system benchmark remained a preprint at the update date.

## Conclusions

The identified literature supports cautious use of AI for specific anatomical variant tasks, with the strongest cluster in dental and cranio-maxillofacial imaging. It does not establish transferable variant awareness across systems or settings. Patient-level reporting, explicit taxonomies, appropriate prevalence-aware metrics, defensible reference standards, external validation, and direct educational outcome studies are needed before broader clinical or curricular claims are justified.
